# Educational Impact of Generalist–Specialist Collaborative Outpatient Training for Medical Students: A Qualitative Study

**DOI:** 10.1002/jgf2.70167

**Published:** 2026-08-16

**Authors:** Koki Nakamura, Masayoshi Oikawa, Tomoo Hidaka, Satoshi Kanke, Koji Otani

**Affiliations:** ^1^ Center for Medical Education and Career Development Fukushima Medical University Fukushima Japan; ^2^ Fukushima Centre for General Physicians Fukushima Medical University Fukushima Japan; ^3^ Department of Cardiovascular Medicine Fukushima Medical University Fukushima Japan; ^4^ Department of Hygiene and Preventive Medicine Fukushima Medical University Fukushima Japan; ^5^ Department of General Internal Medicine and Family Medicine Fukushima Medical University Fukushima Japan; ^6^ Department of Orthopaedic Surgery Fukushima Medical University Fukushima Japan

**Keywords:** ambulatory care, clinical clerkship, family doctor, mini‐CEX, open‐coding, undergraduate medical education

## Abstract

**Background:**

Despite the recognized benefits of specialty outpatient training for medical students, such settings present educational challenges. To address these, our university incorporated generalist support into specialty outpatient education. The present study aimed to explore what medical students learned from this program.

**Methods:**

Fifth‐ and sixth‐year medical students who selected orthopedics or cardiology for their clinical clerkships were enrolled. During training at specialty outpatient clinics, the students conducted preliminary interviews with new patients under generalist supervision and received immediate feedback from the generalists. They then presented the cases to specialists and observed the subsequent examinations, receiving additional feedback. Afterward, they completed a reflection sheet. Text data from the reflections were analyzed thematically using open coding.

**Results:**

Sixty‐four medical students participated. Four categories were generated based on learning from generalists: Efficient information gathering through the synergistic use of open and closed questions; understanding the psychosocial background of the patient and their family through the interpretive model; communication skills to build rapport with the patient; and awareness of the difficulty and importance of balancing patient interviews and chart documentation. From orthopedic specialists, two categories emerged: Proactive approaches emphasizing interviewing and physical examination rather than relying solely on referrals, and insights gained from actual clinical examinations and consideration for patients. From cardiology specialists, two categories were generated: Proactive interviewing and professional perspectives independent of referrals; and explanations that foster patient understanding and co‐creation of treatment plans.

**Conclusion:**

Medical students gained distinct learning experiences from generalists and specialists through generalist‐supported outpatient training.

## 1 Introduction

One of the most significant changes in the healthcare system in recent years has been the dramatic shift from traditional inpatient care to outpatient care [1]. With this transition, the importance of the outpatient setting as a venue for clinical medical education has been emphasized [2]. While approximately 80% of clinical education is conducted in inpatient settings, approximately 80%–90% of actual medical care takes place in outpatient settings [3]. Accordingly, many medical schools around the world have gradually expanded the time allocated to outpatient care [4]. Compared to inpatient care, outpatient care is highly beneficial for medical students in their acquisition of clinical skills such as history taking, physical examination, clinical reasoning, and communication [5, 6].

Despite these needs and benefits, clinical education in outpatient settings has several barriers [7]. In the narrative review by Franco et al., insufficient teaching time was identified as the most common and relevant barrier. Both students and faculty noted a lack of feedback from instructors, while faculty also expressed concerns that the presence of students might disrupt the flow of outpatient care and compromise patient care [8]. Literature on outpatient education is limited and often relies on general practice/family medicine studies. Given the differences between general and specialty outpatient care, findings from general outpatient settings are only partially transferable to specialty outpatient settings [9]. Although Thompson et al. proposed 12 tips for integrating medical students into specialty outpatient care [10], many challenges remain unresolved, and practical educational models that balance teaching and clinical efficiency in specialty settings are lacking [11].

To address this gap, Fukushima Medical University introduced a novel outpatient training model in which generalists provide on‐site educational support within specialty outpatient clinics. This generalist–specialist collaboration enables specialists to maintain clinical efficiency while engaging in teaching and allows students to receive feedback from both perspectives through a single patient encounter. Such a collaborative outpatient training model in a specialty setting is itself innovative, and its educational effects have not yet been clarified. The aim of this study was to explore what medical students learn from generalists and specialists through this collaborative outpatient training model.

## 2 Methods

### 2.1 Study Participants and Setting

This study enrolled fifth‐ and sixth‐year medical students at Fukushima Medical University School of Medicine who chose either orthopedics or cardiovascular medicine for their clinical training between September 2022 and July 2024. Due to limitations in generalist staffing, implementing the program across all departments was not feasible; therefore, the study focused on the two departments that consented to participate. Medical education in Japan is a six‐year program, and fifth‐ and sixth‐year students are in the final stage of pre‐graduation clinical clerkship training. At our institution, systematic lectures in cardiology and orthopedic surgery are delivered in the third year, followed by systematic lectures in family medicine in the first semester of the fourth year. Clinical clerkships begin in the second semester of the fourth year, and required rotations in cardiology, orthopedic surgery, and family medicine are completed by the first semester of the fifth year. Accordingly, all participants had already completed the required lectures and clinical clerkships in these disciplines before selecting orthopedic surgery or cardiology as an elective clinical rotation. The clinical training took place in the outpatient clinics of the Orthopedic Surgery and Cardiology divisions at Fukushima Medical University Hospital. The former division primarily specializes in spine disorders, which require detailed neurological examinations of the upper and lower limbs, including assessments of sensory function, muscle strength, and tendon reflexes. The latter division specializes in valvular disease and cardiac amyloidosis, conditions in which treatment decisions require careful consensus building with the patient and their family, taking into account the patient's age, background, risk factors, and prognosis.

### 2.2 Practicum Content

**FIGURE 1:**
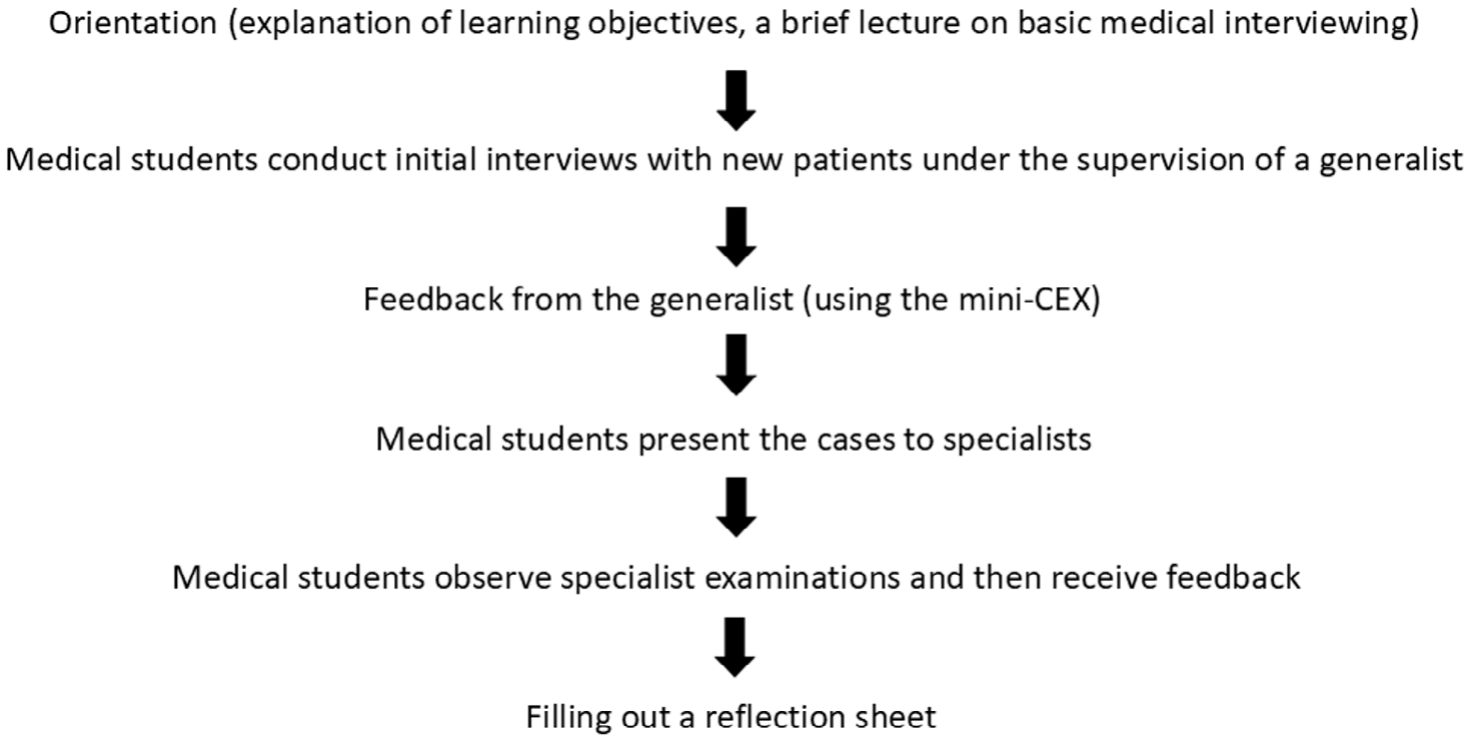
Outpatient training flow legend. While generalists are instructing medical students, specialists can continue providing care for other patients, thereby helping them balance their clinical and educational responsibilities.

The duration of the clinical training was 4 weeks for both orthopedics and cardiology. Outpatient training was scheduled every other Wednesday for orthopedics and every other Tuesday for cardiology. The flow of the outpatient training is shown in Figure 1. First, medical students attended an orientation led by a generalist, which included an overview of the training and a mini‐lecture on OPQRST and “KAKIKAE.” OPQRST is a commonly used framework for history taking, consisting of Onset, Provocation/Palliation, Quality, Region/Radiation, Severity, and Time/Temporal pattern [12]. KAKIKAE is a framework used to elicit patients' interpretive models, referring to how patients understand and make sense of their illness [13]. The acronym is derived from four Japanese concepts: Kaishaku (interpretation: The patient's understanding of the cause, meaning, or reason for the illness and symptoms), Kitai (expectation: The reason for seeking care and hopes regarding future outcomes), Kanjo (emotion: Current feelings and concerns related to the symptoms), and Eikyo (impact: The effects of the illness and healthcare‐seeking behavior on the body and daily life) [13]. Internationally, this framework corresponds to FIFE (Feelings, Ideas, Function, Expectations) [14]. Exploring these interpretive models is considered essential for patient‐centered care [15] and is a required learning objective in undergraduate medical education in Japan [16]. Next, under the supervision of the generalist, the students conducted preliminary assessments of new patients in their respective divisions and documented their findings in the medical records. They then received feedback from the generalist using the mini‐Clinical Evaluation Exercise (Mini‐CEX), an assessment tool designed to observe students' clinical skills in real clinical settings and to provide structured evaluation and feedback [16]. Finally, they presented their findings to the respective specialists, observed the specialists' examinations of the same patients, and received additional feedback.

### 2.3 Data Collection

Medical students completed a reflection sheet at the end of each outpatient training session (Supporting Information 1 for the reflection sheet). The sheet included open‐ended questions to facilitate reflection on their clinical experiences. The following responses were analyzed: “What did you learn most from the generalists' feedback?” and “What did you learn most from the specialists' feedback?” Reflective writing has been recognized as a valuable source of qualitative data for understanding learners' experiences in health professions education, as demonstrated in qualitative meta‐syntheses [17]. In addition, reflective writing has been shown to promote the development of clinical skills and communication competencies, making it well suited to outpatient clinical training. Because reflection is effective when conducted soon after an experience, data were collected at the end of each outpatient training session.

### 2.4 Data Analysis

Responses collected were analyzed using open coding, a well‐established qualitative method in medical education research [18]. The analysis was conducted separately for content related to generalists and specialists. Concise labels were assigned to segments of data according to their meaning. Through stepwise abstraction and categorization, the dataset was systematically organized to enable comprehensive analysis. Focusing on the educational effects of the practicum, we extracted segments related to the changes and growth in the students after the training. Regarding the relationship between the researchers and the participants, in this study, the generalist (KN), orthopedic surgeons, and cardiologists were involved in supervising students during the outpatient training. However, the researchers were not involved in the summative assessment of the overall clinical clerkships in each department. Methodological triangulation and researcher triangulation were conducted to increase the reliability of the qualitative analysis. For methodological triangulation, generalist‐related findings were cross‐checked against corresponding Mini‐CEX records, whereas specialist‐related findings were verified against generalists' observation records of specialist feedback sessions. For researcher triangulation, the initial coding was conducted by the generalist who supervised students in the clinical setting (KN). To minimize potential interpretive bias, another generalist (SK), who was not involved in the outpatient training, independently reviewed and verified the coding. The entire analysis process was supervised by an experienced qualitative researcher (TH) who had no involvement in the outpatient training. TH holds doctoral degrees in psychology and medicine and has an established record of qualitative research in medical education. All researchers collaboratively reviewed the abstraction process, the appropriateness of labels and thematic terms, and possible alternative interpretations of the data. Data saturation was considered achieved when no new codes were identified through this iterative review.

### 2.5 Ethical Considerations

This study was approved by the Research Ethics Committee of Fukushima Medical University (Approval No. 2022–166). Written informed consent was obtained from all participating students via the reflection sheet. No written consent has been obtained from the patients as there is no patient‐identifiable data included. During the orientation, the researchers provided each student with an individual explanation using an information sheet approved by the institutional ethics committee. Participation was voluntary, and non‐participation did not affect grades or clerkship evaluation; this was clearly stated in both the information sheet and the reflection sheet (Supporting Information 1 for the reflection sheet).

## 3 Results

Sixty‐four medical students participated in this study. The characteristics of the participants are presented in Table 1. Open coding generated 64 initial labels from the narrative data, which were then grouped into 13 subcategories and finally summarized into eight categories. All data are available in Supporting Information 2 for the dataset.

**TABLE 1:** Baseline characteristics of study participants.

Average age (years)	24
**Sex**	
Male	46 (72%)
Female	18 (28%)
**Outpatient training site**	
Orthopedic Surgery division	33 (52%)
Cardiology division	31 (48%)

### 3.1 Categories Related to Learning From the Generalists

Four categories were generated (Table 2).

**TABLE 2:** Categories related to learning from generalists.

Category	Subcategory	Code	Description of the study participants
Efficient information gathering through the synergistic use of open and closed questions	Techniques for efficiently collecting OPQRST and interpretive model information through the use of open questions	Approaches to identify information on OPQRST and *kakikae* from the open question narrative and to ask additional questions about the missing parts	I learned that patients' responses to open questions often contain elements of OPQRST and *kakikae*, and it is important to identify and extract them. I also learned that it is wise to ask additional questions to fill in any potentially missing information.
		Conscious listening for interpretations, expectations, feelings, and influences expressed in responses to open questions	It is important to listen carefully to the patient's responses to open questions, as they often express their interpretations, expectations, feelings, and influences.
		Efficient and high‐quality information collection through organizing OPQRST and *kakikae* elements within patients' free narratives	Patients often speak freely about OPQRST and *kakikae* elements, so organizing their narrative helps avoid redundant questioning and preserves the quality of information.
		Designing a medical interview that makes use of the patient's free narrative while supplementing missing OPQRST elements with closed questions	It is difficult to listen to the patients in the order of OPQRST, so the missing information will be filled in by closed questions as the patients talk.
		Picking up information from the patient's narrative to save time and maintain their comfort in speaking	By picking up information naturally during the conversation, the interview can be shortened, allowing patients to speak at their own pace.
		Strategies to conduct an efficient and high‐quality medical interview using information obtained from open questions	OPQRST elements, etc., do not need to be asked independently, and if *p* can be addressed through open questions, it can be omitted; by combining the elements that can be asked together, a higher‐quality interview can be conducted in a shorter amount of time.
		OPQRST preparation for a smooth medical interview	I thought that preparing the OPQRST framework in the chart or notes beforehand and filling it in by asking closed questions as the patient spoke would help reduce missed questions and make the interview flow more smoothly.
		Awareness of a flexible listening approach that organizes OPQRST elements without strict adherence to their order	I adhered to the OPQRST order to avoid missing anything, but even if the order goes back and forth, I will be able to listen more flexibly and organize the information.
		Recognition of the importance and difficulty of extracting OPQRST elements from patients' free narratives	I learned that real patients do not always answer the OPQRST in order, and although it is difficult, it is also important and difficult to filter out each element in the flow of the conversation.
	Limitations of open questions and the importance of closed questions to compensate for them	Design a medical interview to supplement information not obtained through open questions, and to confirm negative findings through closed questions	Ask closed questions on topics that could not be covered by the open questions. It is also important to confirm negative findings, such as the absence of chest pain or shortness of breath.
		Importance of using open and closed questions in real‐world clinical practice	Open questions are important, but it is also important in the clinical setting to narrow down the possibilities using closed questions.
		Flexible interviewing without adhering to OPQRST, and an awareness of actively drawing out unspoken information	I was focused on applying the OPQRST framework, but I wanted to develop the ability to ask questions tailored to the symptoms. I also came to strongly realize the importance of eliciting information that patients do not mention themselves, such as dizziness or edema.
		Techniques for interviewing patients to elicit information they may not voluntarily provide, while keeping OPQRST in mind	Interviews keeping OPQRST in mind. Elicit what the patient has not yet voluntarily said (what they think is not related to this illness).
		Awareness of the limitations of open questioning due to the patient's selective disclosure of information	I learned that the information obtained through open questions is subject to whatever the patient chooses to disclose, and that sometimes the desired information is not obtained.
		Strategies to elicit unrecognized symptoms in patients who do not have any chief complaints, using closed questions	It would have been better if, in the case of a patient who did not have a chief complaint, I had used closed questions to uncover points the patient was unaware of, rather than relying solely on open questions.
		The importance of being able to organize information through the use of closed‐ended questions to identify accompanying symptoms	I learned that it is sometimes better to ask more closed questions when asking about accompanying symptoms. In clinical practice, patients often provide information in fragments, so it is important to be able to gather and organize these details effectively.
		The difficulty of open questioning and the need for a focused interview aimed at differential diagnosis	I found it difficult to hear information through open questioning. I learned that I need to independently consider the differential diagnosis and focus on the key points I need to ask.
Understanding the psychosocial background of the patient and their family through the interpretive model	Holistic understanding of the patient, including their psychosocial background, through the interpretive model	Conscious questioning to understand the interpretive model	I learned a lot about how to listen for interpretive models, something I had never really thought about before.
		Interview and treatment awareness to understand the patient's problems and thoughts while advancing the differential diagnosis process	While the physician conducts differential diagnosis during the examination, it is important to recognize that each patient has unique thoughts and concerns related to their symptoms, and to integrate these into the interview and treatment process.
		Focus on daily life problems in orthopedic practice	In orthopedics, it is important to ask patients what they need help with in their daily lives.
		A holistic viewpoint that interprets *kakikae* not in a conventional way, but in a holistic way that includes personality and character.	It is important to understand the patient's unique personality and character as a whole, rather than just following a interprets.
		Awareness of the importance of listening carefully to the individual patient's complaints through *kakikae*	I realized the importance of listening to the individual patient's complaints by asking about *kakikae*.
		Inferring the impact on daily life based on how symptoms are expressed, and finding natural ways to draw out underlying problems	I learned that the language patients use to describe their symptoms can reveal the impact on their daily lives, and by requesting further details, I can smoothly understand the challenges they face throughout the conversation.
		Shift in mindset from a disease‐centered perspective to a medical interview that considers the background of the patient and their family	I learned how difficult it is to understand the social background and economic situation of the patients. In medical interviews, my mind is inevitably occupied with disease‐related issues, so I would like to learn how to speak in a way that allows me to focus more on the patients and their families.
	Awareness of the possibility that interpretive models may differ between patients and their families	Awareness of the need to reflect on preconceived assumptions and to listen carefully to individual models of interpretation	I regretted not being able to explore each person's perspective more deeply, as I had preconceived notions that family members and patients would share the same concerns or expectations regarding the effects of treatment when I asked about their interpretive models.
		Approaches to facilitate closing by listening separately to the interpretations and expectations of the patient and their companion	If the patient is accompanied by a companion, the closing process will be smoother by interviewing them separately, as their interpretations and expectations may differ.
		Importance of an attitude to confirm agreement or disagreement between the family's and the patient's wishes	It is important to confirm whether the family's wishes align with, or differ from, those of the patient.
Communication skills to build rapport with the patient		Responding according to the patient's personality	It is best to adjust your approach and tone to suit the patient's personality.
		Consideration to respect spontaneous narratives and avoid giving the impression of asking the same questions	If the patient spontaneously gives us information (OPQRST, *kakikae*, etc.), we do not need to repeat the same questions (to avoid giving the impression that we are repeating questions, rather than to avoid missing information).
		Building communication through empathy for both difficulties and joys	To build effective communication, it is important to empathize with both the difficulties and the joys.
		Techniques that promote trust and smooth communication by using the patient's own words verbatim	The patient's chief complaint should be documented in the patient's own words, and using those words can help build trust and facilitate smooth communication.
		Recognition of the importance and difficulty of interviewing patients along the flow of their narratives	I learned the difficulty and importance of interviewing patients according to the flow of their speech, not my own flow.
		Techniques to deepen the relationship by incorporating not only closed questions but also interpretive models and open questions	I felt that incorporating not only closed questions but also open questions and *kakikae* could improve the doctor‐patient relationship and facilitate a smoother examination.
		Closing touches that make patients feel their concerns have been acknowledged by summarizing	By summarizing and then closing, the patient feels that they were understood.
		Importance of devising ways to obtain information efficiently in a short time without repeating the same questions	I asked the same questions over and over again, but I thought there could be a way to obtain the same information more efficiently by avoiding this.
		Awareness of the importance of maintaining eye contact even during electronic medical record entry	Unlike interviewing patients while taking handwritten notes, conducting the interview by directly typing into the electronic medical record was easier in terms of input, but I had to consciously maintain eye contact with the patient, or I would almost forget.
Awareness of the difficulty and importance of balancing patient interviews and chart documentation		Organizing information with charting in mind while acknowledging the practical challenges involved	I thought it was important to listen carefully while gathering the information needed for documentation. The interview went smoothly, but I found it challenging to organize the information effectively in the medical record.
		Awareness of the importance of carefully documenting findings as heard, without depending solely on a template	I thought it was important to write detailed and thorough descriptions of the findings that I heard, rather than simply following a template.

*Note:* OPQRST stands for: Onset, provocation/palliation, quality, region/radiation, severity, and time/temporal pattern [12]. The *kakikae* includes: *kaishaku* (interpretation), *kitai* (expectation), *kanjo* (emotion), and *eikyo* (impact) [13].

#### 3.1.1 Efficient Information Gathering Through the Synergistic Use of Open and Closed Questions

The participants found that information related to OPQRST and interpretive models was scattered in the free responses to open questions. They learned that by identifying this information and using closed questions to fill in missing details, they could efficiently obtain high‐quality information in a short period of time. They also recognized the limitation of open‐ended questioning—namely, its inability to elicit symptoms that patients are either unaware of or consider unrelated to their chief complaint. They learned that, in such cases, it is necessary to actively seek out diagnostically relevant information using closed‐ended questions.

#### 3.1.2 Understanding the Psychosocial Background of the Patient and Their Family Through the Interpretive Model

The participants learned the importance of attending not only to diagnostic information but also to the psychosocial background of patients, such as their concerns and anxieties about daily life. They also recognized that when family members are present during medical interviews, the interpretive models of the patient and their family may differ, potentially affecting treatment decision‐making.

#### 3.1.3 Communication Skills to Build Rapport With the Patient

The participants learned that respectfully attending to a patient's free narrative facilitates more open communication. They realized that repeated questioning unnecessarily lengthens the medical interview but may also undermine trust by suggesting inattentiveness. In addition, they noticed that summarizing key points at the end of the interview not only helps organize information for the medical staff but also reassures the patient that their concerns have been accurately understood. The participants further learned other practical communication strategies, such as rapport building, empathic responses, and the use of eye contact.

#### 3.1.4 Awareness of the Difficulty and Importance of Balancing Patient Interviews and Chart Documentation

The participants recognized the challenges and significance of documenting patient information while actively listening during interviews. Although templates were useful, the participants also came to understand their limitations.

### 3.2 Categories Related to Learning From the Orthopedic Specialists

Two categories were generated (Table 3).

**TABLE 3:** Categories related to learning from orthopedic specialists.

Category	Subcategory	Code	Description of the study participants
Proactive approaches emphasizing interviewing and physical examination rather than relying solely on referrals	A professional perspective on the diagnosis through medical interview and physical examination	Recognition of the importance of a more detailed symptom interview	(The specialist) was more detailed when asking about symptoms.
		Recognition of the importance of carefully identifying site and symptoms without taking the patient's complaints at face value	Do not accept the symptoms complained of by the patient as they are. Ask for details, keeping in mind that even for the same body part, the perceived location may vary between individuals.
		Recognition of the importance of being aware of the sequence of events from symptoms to diagnosis	I wanted to accurately identify various signs and keep track of the sequence of events leading to their diagnosis.
		Importance of understanding positive and negative findings for differentiation	I felt it was important to list the differential diseases and take positive and negative findings to aid in differentiation.
		Recognition of the importance of approaching the diagnosis through a careful interview and physical examination	Many diagnoses can be made not only through imaging but also by thorough interviews and physical examinations. The patient's reflexes and other symptoms should be examined one by one.
		Physical examination with a clear sense of purpose	I realized the importance of being clear about what I wanted to observe during the physical examination and the purpose behind it.
		Accurate diagnosis through physical examination, with consideration of potential disabilities suggested by the patient's complaints	I learned that I can make an accurate diagnosis by conducting a physical examination while anticipating what is impaired, as well as how it is impaired, from the patient's complaints.
		Awareness of the specialist's deep knowledge and important points in conducting interviews and examinations	(Specialists) possess knowledge and expertise that are entirely different from my own. I realized that there were important points I needed to pay attention to and not overlook.
	A proactive attitude to seek information independently without relying on referral sources	Recognition of the importance of obtaining physical findings from scratch, without relying on the previous physician's diagnosis	I learned that it is important to conduct a thorough physical examination from the ground up, because the previous doctor's diagnosis is not always 100% correct.
Insights gained from actual clinical examinations and consideration for patients	Discovering insights into medical examinations and procedures that could only be gained through interaction with real patients	Valuable learning experience of abnormal findings gained through actual neurological examinations	Since we had only practiced reflex examinations on healthy individuals, we hadn't had many opportunities to observe actual cases of hyperreflexia or hyporeflexia. It was a great opportunity for me to observe and perform MMT and neurological examinations in a real clinical setting.
		Experience learning the proper techniques of basic clinical procedures (tendon reflexes, MMT, injections)	I was able to learn basic clinical techniques, including tendon reflex testing, MMT, and the administration of pain relief injections.
	Physical examinations that do not burden patients with lower limb or back problems	Recognition of the difficulty and importance of performing a physical examination while taking into consideration the patient's movements	I learned a lot through actually performing physical examinations—e.g., that the patient's movements didn't align with the examination I intended to conduct, or that I asked them to move unnecessarily.
		Neurological examinations with less patient strain	Neurological examination while minimizing changes to the patient's posture as much as possible.

#### 3.2.1 Proactive Approaches Emphasizing Interviewing and Physical Examination Rather Than Relying Solely on Referrals

The participants learned the importance of systematic interviews and physical examinations by observing consultations conducted by specialists. They realized that predicting the lesion site through a detailed history and carefully confirming both positive and negative findings through a focused physical examination can lead to an accurate diagnosis. They also realized the importance of actively gathering information on their own, rather than simply accepting the referring physician's diagnosis.

#### 3.2.2 Insights Gained From Actual Clinical Examinations and Consideration for Patients

The participants learned from identifying abnormal findings in actual patients, rather than being limited to normal findings in peer simulations. They also realized the importance of structuring the physical examination to minimize the burden on patients who have difficulty moving due to problems with their legs or back.

### 3.3 Categories Related to Learning From the Cardiology Specialists

Two categories were generated (Table 4).

**TABLE 4:** Categories related to learning from cardiology specialists.

Category	Subcategory	Code	Description of the study participants
Proactive interviewing and professional perspectives independent of referrals	Professional perspectives in interviewing, consultation, and diagnosis	Professional discomfort regarding differential diagnoses and interpretation of clinical findings	I realized that a professional perspective reveals different insights, such as in differential diagnoses and unusual findings.
		Targeted interviews and physical examinations with consideration of subsequent testing and treatment	(The specialist) conducted a more detailed interview and carefully considered the examination and treatment.
	Detailed interview regarding daily activities and medications	Eliciting specific information about shortness of breath during stair climbing	I realized that it is important to ask detailed questions about shortness of breath during stair climbing, such as how many flights of stairs cause breathlessness.
		Necessity of interviewing regarding activities of daily living	I should have asked the patient about his activities of daily living, such as whether he could walk.
		Confirmation of oral medications with consideration of their effectiveness and impact on improving medical conditions	I felt that it is very important to check oral medications because some of the currently prescribed medications may be ineffective, or even have adverse effects on the patient's condition.
	Proactive attitude of gathering information independently rather than relying solely on the referring source	Importance of thoroughly confirming key findings and edema beyond the diagnosis provided by the referral source	In addition to the diagnosis from the referral source, the specialist carefully examined important findings and edema, which I felt was crucial to ensure an accurate diagnosis.
		The importance of gathering and organizing information on your own without relying on referral letters	I realized the need to gather information on my own, rather than relying solely on the referral letter from the previous doctor. I also learned the importance of staying calm and organizing the information effectively.
Explanations that foster patient understanding and co‐creation of treatment plans	Techniques for providing explanations that are understandable and convincing to the patient	Recognizing the importance of clear explanations and careful word choice to ensure patient understanding	I realized that I particularly lacked the skill to explain symptoms clearly, using images and rephrasing them into words that patients can easily understand.
		Careful explanation of possible complications	I thought it was important to carefully explain not only about the disease, but also about possible complications associated with treatment and testing.
		Explanation of medical condition supported by evidence from test results	He (the specialist) was explaining the patient's condition, showing the test results as appropriate, and the patient seemed to be very satisfied with the explanation. I thought such visible evidence was important.
	Mutual decision‐making attitude based on each patient's medical condition and schedule	A collaborative approach to developing treatment plans and schedules with patients and their families	It was impressive to see how the specialists worked together with patients and their families to develop a treatment plan and schedule.
		The importance of determining treatment timing and individualized plans based on the severity of symptoms and conditions	Observing how the specialist determined the appropriate timing of treatment based on the severity of symptoms and the patient's condition made me realize once again the importance of tailoring treatment plans to each individual patient.
		Enhancing patient understanding through explanation of the current condition and proposed treatment plan	The flow was to first accurately inform the patient of the current situation and then clarify the future course of action, so the patient seemed to have a very good understanding of the situation. I would like to be able to do the same.

#### 3.3.1 Proactive Interviewing and Professional Perspectives Independent of Referrals

The participants learned more in‐depth interviewing techniques from specialists that extended beyond symptoms to include daily life and medication use. They also realized the value of independently verifying information rather than depending entirely on prior diagnoses.

#### 3.3.2 Explanations That Foster Patient Understanding and Co‐Creation of Treatment Plans

The participants learned patient‐friendly communication techniques from specialist consultations. They also recognized the importance of personalized shared decision‐making tailored to each patient.

## 4 Discussion

The present study demonstrated that learning from generalists included the following four categories: “efficient information gathering through the synergistic use of open and closed questions,” “understanding the psychosocial background of the patient and their family through the interpretive model,” “communication skills to build rapport with the patient,” and “awareness of the difficulty and importance of balancing patient interviews and chart documentation.” Previous research has reported that communication skills, medical record documentation, history taking, and understanding of psychosocial factors can be learned through general practice/family medicine training [19]. The novelty of our study is that learning comparable to that reported in general practice/family medicine training was also achieved in university hospital specialty clinics. However, opportunities for medical students to participate in general practice/family medicine training remain inadequate worldwide [19, 20]. In this context, involving generalists in specialty clinical training may enhance the educational impact of general/family medicine for medical students.

Interestingly, the participants learned that proactively gathering information rather than relying on referrals is an important aspect of professionalism in both orthopedic and cardiology specialty training. Given that referral information is often insufficient for accurate clinical decision‐making [21], this represents a unique learning opportunity that cannot be encountered during training at referring institutions. In orthopedics, the participants learned that physical examinations involve not only identifying abnormal findings but also arranging the examination to reduce the burden on patients. The participants also emphasized the importance of maintaining professionalism and courtesy when examining patients, which aligns with previous research showing that medical students learn to engage with real patients with respect and professionalism through hands‐on experience [22]. In cardiology, the participants learned how to provide explanations that foster patient understanding, as well as the importance of collaboratively developing treatment plans with patients. Learning shared decision‐making is essential for medical students, and various educational approaches have been developed to support this [23]. In specialty outpatient clinics, in particular, students can observe shared decision‐making in real time, which provides a valuable learning experience. This is particularly important because such opportunities are limited in inpatient settings, where diagnoses and treatment plans are usually already established.

This study has several limitations. First, it was conducted at a single university, making it difficult to generalize the results beyond this setting. Second, the reflection sheets were self‐reported by students and may be subject to bias. Students may have expressed overly positive views out of gratitude or respect for the supervising physicians. Third, because this specialty outpatient training was a one‐time educational intervention, its long‐term impact remains unclear. In the future, it will be necessary to repeatedly conduct specialty outpatient training to longitudinally assess the students' growth. Fourth, a priming effect from the generalist's mini‐lecture delivered prior to the clinical encounters cannot be completely excluded, although the students' reflections suggested learning shaped by actual patient encounters and supervisor feedback rather than mere knowledge recall. Fifth, reflective writing may be limited compared with individual interviews or focus groups in terms of confirming context, probing participants' intentions more deeply, and exploring emerging themes. Future studies incorporating interview methods may provide a more comprehensive understanding of students' experiences.

This generalist–specialist outpatient education model may be applicable to training in specialties beyond cardiology and orthopedics. Gradually expanding the target specialties would allow further evaluation of both the feasibility of implementation and the educational impact, while taking into account the limited generalist resources and the sustainability of the model.

## 5 Conclusion

The present study demonstrated that medical students acquired practical insights from both generalist and specialist perspectives through specialist outpatient clinical training supported by generalists. These findings provide important implications for the use of outpatient clinical settings as learning environments for medical students.

## Author Contributions

**Koki Nakamura:** conceptualization, investigation, writing – original draft, methodology, formal analysis, project administration, data curation, visualization. **Tomoo Hidaka:** writing – review and editing, methodology, supervision. **Satoshi Kanke:** formal analysis, writing – review and editing. **Koji Otani:** resources, writing – review and editing, supervision. **Masayoshi Oikawa:** resources, writing – review and editing.

## Conflicts of Interest

The authors declare no conflicts of interest.

## Supporting information


**Supporting Information: 1** Reflection Sheet for Outpatient Preliminary Assessment Training.

**Supporting Information: 2** Supporting Information.

## Data Availability

The data that supports the findings of this study are available in the Supporting Information 1 of this article.
