# Supplementary material for: Educational Impact of Generalist–Specialist Collaborative Outpatient Training for Medical Students: A Qualitative Study
Source: J Gen Fam Med. 2026 Aug 16;27(5):e70167. doi: 10.1002/jgf2.70167 (PMC13478716; doi:10.1002/jgf2.70167)
Supplement: Supplementary file 1 [file JGF2-27-e70167-s001.docx]

Supplementary Material 1

**Reflection Sheet for Outpatient Preliminary Assessment Training**

**Date:** ________ Year ____ Month ____ Day (________ Day of week) **Age:** ________ **Sex:** □1. Male / □2. Female

**Clinical Site:** □1. Orthopedic Surgery □2. Cardiology

**Study Title: "Educational Impact of Generalist–Specialist Collaborative Outpatient Training for Medical Students: A Qualitative Study"**

Regarding the use of information from this reflection sheet and the Mini-CEX sheet for the research study named above:

[ ] **I agree** [ ] **I do not agree**

Participation in this study is entirely voluntary. Choosing not to participate will not result in any disadvantage regarding your clinical training or grades. Please feel free to provide honest feedback, as this sheet will be handled separately from your academic performance evaluations.

**1. How many times have you conducted a medical interview/history taking with a patient during your Clinical Clerkship (BSL) so far? (Exclude simulated patients) [Select one]**

1. 5 times or less
2. 6–10 times
3. 11–15 times
4. 16–20 times
5. 21 times or more

**2. How often do you think the experience of "conducting a medical interview/history taking with a patient" is necessary during BSL? (Exclude simulated patients) [Select one]**

1. Once a week or more
2. About once every two weeks
3. About once a month
4. Once every two months or less

**3. What did you learn most from the feedback provided by the Generalist (General Physician)?** *Verbalize your learning by writing it down!* **Important: Do not include any patient-identifiable information.**

**4. What did you learn most from the feedback provided by the Specialist (Organ-specific Specialist)?** *Verbalize your learning by writing it down!* **Important: Do not include any patient-identifiable information.**
