# Supplementary material for: Educational Impact of Generalist–Specialist Collaborative Outpatient Training for Medical Students: A Qualitative Study
Source: J Gen Fam Med. 2026 Aug 16;27(5):e70167. doi: 10.1002/jgf2.70167 (PMC13478716; doi:10.1002/jgf2.70167)
Supplement: Supplementary file 2 [file JGF2-27-e70167-s002.docx]

Supplementary Material 2

**Learning from generalists**

I learned that when patients speak freely in response to open questions, their narratives often already contain elements of OPQRST and information relevant to kakikae. Therefore, it is important to identify and organize the information within the patient’s story, and then ask focused follow-up questions only to elicit any potentially missing information. I realized that this approach leads to a smoother interview.

I learned that during the open questioning phase, patients often naturally express their interpretations, expectations, emotions, and the impact of symptoms on their lives. It is important to be mindful of these aspects when listening to the patient.

I received feedback on my use of open and closed questions, as well as my ability to identify negative findings—things I had previously done unconsciously. This made me reflect on my interviewing practices. I had not previously been particularly aware of how to explore a patient’s explanatory model, so this was especially educational for me.

Receiving objective feedback was very helpful. I interviewed three patients, and was able to identify specific points for future improvement. Although I was nervous, carrying out the interview firsthand facilitated a more memorable learning experience.

I learned that adjusting my posture and tone to suit the patient’s personality helps build rapport. I also thought that writing down OPQRST in advance on a chart or memo, and then filling in missing information with closed questions while listening to the patient, could reduce omissions and make the interview flow more smoothly.

I came to understand how difficult a patient’s social and economic circumstances can be. During medical interviews, I tend to place too much emphasis on the disease itself, so I would like to develop a communication style that allows me to engage with the patient and their family as individuals.

Receiving feedback immediately after each encounter helped me reflect while the experience was still fresh. The feedback included both strengths and points for improvement, which I feel will influence how I approach future practice. I also learned the importance of considering the patient’s perspective from a generalist’s point of view.

As patients freely talk about information related to OPQRST and kakikae, organizing what they say allows me to avoid asking the same questions twice without compromising the quality of the information.

I learned that it is important to organize essential information for medical record documentation while interviewing. Although the interviews themselves went smoothly, I struggled when summarizing them in their charts.

During medical interviews, while the physician progresses through a differential diagnosis, each patient has different thoughts and concerns depending on their symptoms. I learned that being aware of this during consultations is important for enhancing both the interview process and treatment.

Information that patients provide spontaneously, such as OPQRST or kakikae, does not need to be asked again. Avoiding repetition is more important than ensuring nothing is missed, as repeating questions may give the impression of asking the same thing twice.

When eliciting the patient’s explanatory model, I realized that I had assumed that the patient and their family shared similar concerns and expectations regarding the illness and treatment. This prevented me from exploring individual perspectives in depth. I reflected that I should have made a greater effort to listen attentively to the patient and their family within the time available.

I learned the importance of incorporating the perspective of accompanying family members into the conversation. Having a structured approach helps prevent omissions, and empathizing with the emotions of both the patient and the family is useful.

I felt that it would have been better if I had also listened to the family members’ accounts and completed the medical records more thoroughly.

When a patient is accompanied by their family, the patient’s interpretations and expectations may differ from those of their family. Asking them separately makes closing the interview smoother. I also learned not to be overly fixated on asking OPQRST strictly in the order listed; information can be organized organically as the conversation progresses.

Especially in orthopedic consultations, it is important to ask what aspects of daily life the patient finds difficult. Since it can be hard to strictly adhere to the OPQRST format, missing information can be supplemented with closed questions during the conversation.

I learned that effective communication involves empathizing with both the patient’s difficulties and positive experiences. It is also important to confirm whether the patient’s and family’s hopes are aligned or not.

I learned the importance of conducting a thorough interview while also adapting to the patient’s pace.

By identifying what key points are most relevant to the patient’s symptoms, it becomes easier to summarize their narrative.

Time management is important. Gathering information naturally from the patient’s narrative allows the interview to proceed smoothly and efficiently. I realized that I hadn’t asked the patient about their allergy history or the timing of their symptoms.

When open questions do not elicit certain details, it is important to follow up with closed questions. Confirming negative symptoms (e.g., absence of chest pain or dyspnea) is also essential. Even if there is no clear chief complaint, there is always a reason why the patient is visiting the clinic.

By writing the chief complaint in the patient’s own words and using their language when communicating, trust and smoother interaction were established. While open questions are important, narrowing down with closed questions is also necessary in real clinical practice.

I learned both the difficulty and importance of following the patient’s narrative rather than imposing my own interview structure. I appreciated receiving assistance when I was unsure how to respond.

Clarifying the patient’s coping behaviors is important. Also, it is advisable to avoid any redundancy by not asking for the same information twice if it has already been elicited through open questioning.

I found it challenging to link symptoms to the potentially affected nerves during the interview because it was easy to become confused.

I realized that it is important not just to follow the template but to document detailed findings based on what was actually heard. Incorporating both closed and open questions—including eliciting the patient’s kakikae—improves the doctor–patient relationship and makes the consultation smoother.

I became so focused on applying OPQRST that I lost flexibility. I want to learn how to tailor my questions to the patient’s specific symptom pattern. I also realized the importance of eliciting symptoms such as dizziness or edema, which patients may not mention spontaneously.

When kakikae, it is important to understand the patient’s personality and character as a whole rather than strictly following a structured format. I also learned that patients who tend to follow medical instructions closely may be more prone to paternalism, so I should be cautious.

I learned approaches on how to respond to patients who are frustrated due to long waiting times.

I found that summarizing the interview before finishing helps the patient feel that they have been truly heard.

I learned to conduct interviews while being conscious of OPQRST, and to elicit information that the patient may not volunteer because they do not think that it is related to their illness.

I realized that it is not always necessary to ask about the various OPQRST elements separately. If the patient’s narrative already covers multiple components, I can consolidate them and conduct a more efficient and higher-quality interview in less time.

Through kakikae, I recognized the importance of listening carefully to each patient’s individual concerns.

I gained insight into practical methods for conducting medical interviews.

I learned to listen empathetically and to use open questions, focusing not only on the computer but also on observing the patient.

I was reminded of the importance of providing a brief one-minute summary at the end of the interview. I also learned that information obtained through closed questions may be influenced by what the patient chooses to present, and may not always reflect what I am trying to elicit.

For patients who do not present with a clear chief complaint, it would have been better to use closed questions to help them recognize symptoms they had not been aware of.

I learned that it can be useful to use more closed questions when asking about associated symptoms. In clinical practice, information often comes in fragments, so the ability to gather and organize these pieces is necessary.

I learned that, by paying attention to the patient’s own words when describing their symptoms and exploring how these affect daily life, I can understand more easily what difficulties the patient is experiencing.

I learned that real patients do not describe their symptoms in the order of OPQRST. It is important—and challenging—to identify the relevant elements from the natural flow of conversation.

I found it difficult to collect information using open questions. I realized the importance of first establishing possible differential diagnoses and then asking focused questions.

I learned the importance of asking patients what they expect from their consultations.

I learned that when the clinical course is long, the timeline can often become disorganized, so it is important to listen carefully and reconstruct it.

I repeated questions unnecessarily, and realized that avoiding repetition allows the same information to be obtained in a shorter length of time.

I forgot to summarize my findings at the end of the interview. Unlike interviewing while taking handwritten notes, interviewing while simultaneously entering data into a computer required additional effort to maintain eye contact and remain engaged with the patient.

**Learning from orthopedic specialists**

When asking about pain (e.g., “my lower back hurts”), I realized that I still lack skill in clarifying the precise location and characteristics of the pain. I also learned about the value of not assuming the previous physician’s diagnosis is always correct, and instead performing a thorough physical examination from the beginning.

Previously, I had mainly practiced reflex examination on healthy individuals, so I had had few opportunities to actually observe hyperreflexia or diminished reflexes. Observing and performing MMT and neurological examinations during the session was a valuable experience. Conducting these after the interview also helped me understand the overall flow of clinical encounters.

The questioning about symptoms was more detailed than what I had been used to. I learned to think about treatment first and then consider what information I need to gather. I was able to observe clinical practices that do not appear in OSCE or national exams, which was very educational.

I would like to become able to not only elicit various clinical signs accurately but also to integrate these signs into a coherent diagnostic process.

I learned not to take the patient’s description of symptoms at face value. Even if patients use the same term, the area they have in mind may differ from person to person. I realized the need to ask for clarification in detail.

I learned to perform each examination with a clear sense of purpose, and to consider all possible diseases that could explain the symptoms.

I learned that diagnosis can often be made based on medical history and physical examination alone, even without imaging. It is also important not to skip steps such as checking reflexes.

I realized that bedside learning is about grasping the clinical checkpoints that are essential in real practice. I learned the importance of learning through hands-on physical examination.

I learned how to evaluate whether symptoms and findings match, and when they do not, to identify what additional information may be missing.

It is important to conduct the interview while engaging in clinical reasoning, and to consider the possible lesion level while obtaining physical findings.

Performing physical examinations was more difficult than expected. Sometimes the patient’s movements did not match what I intended, and I inadvertently asked them to move unnecessarily, which I only realized through hands-on experience.

I learned that it is important to be aware of what to assess and why during a physical examination. Listing differential diagnoses and carefully obtaining both positive and negative findings is essential to distinguish between them.

Low back pain alone is not specific enough. It is necessary to clarify the exact anatomical location while simultaneously considering possible underlying diseases. I also practiced techniques such as eliciting reflexes.

I realized that my previous knowledge was somewhat superficial. There are key clinical points that must not be overlooked.

I learned that combining neurological examination findings allows for a certain degree of diagnostic clarity.

I learned to continue clinical reasoning during the interview by eliciting negative symptoms and conducting system-based questioning.

I reviewed the proper approach from taking medical histories to performing physical examinations, as well as the mindset needed for each.

I learned how to determine neurological lesion levels, as well as how to collect the necessary physical findings.

I learned that physical examination becomes more accurate when conducted while predicting potential impairments based on the patient’s reported symptoms.

I was able to learn essential clinical techniques such as tendon reflex testing, MMT, and how to administer analgesic injections.

I learned that neurological examination is conducted for the purpose of identifying the cause of the patient’s symptoms.

I learned the importance of minimizing changes in the patient’s position during neurological examination.

**Learning from cardiology specialists**

I learned how to ask about accompanying symptoms and how to perform physical examinations in cardiovascular cases. I realized in particular that I need to improve my ability to explain symptoms and findings using easy-to-understand language and visual materials such as images.

It is ideal to identify specific symptoms during the interview.

In aortic regurgitation, I learned to pay attention to bounding pulses, rapid pulses, valve prolapse, and high-pitched diastolic murmurs.

I was impressed by how the physician discussed treatment plans together with both the patient and their family. I learned the importance of thoroughly explaining not only the disease itself, but also possible complications associated with treatments and tests.

I felt that having a specialized perspective enables more effective recognition of subtle abnormalities and differentiation of conditions more effectively.

I reaffirmed that treatment plans need to be tailored to each patient, taking into account symptom severity and the patient’s overall condition. Drawing out the patient’s narrative remains important.

When asking about dyspnea on exertion, it is helpful to ask specifically how many flights of stairs lead to the patient becoming short of breath.

I reflected that I should have taken a more thorough family history without focusing solely on heart disease. I also realized the importance of reviewing current medications, as some may have little benefit for the patient’s condition or could even have adverse effects.

I learned that mitral regurgitation can lead to cardiac enlargement, pulmonary hypertension, and atrial fibrillation if not surgically treated in a timely manner.

Asking about symptom frequency, as well as how long they were absent, can improve assessment accuracy.

I learned the importance of not relying solely on a patient’s referral letter, but also carefully checking for additional physical findings, such as edema, to avoid missing important diagnoses.

The interview questions were more specific and geared toward planning examinations and treatment.

Diabetic patients may not show clear subjective symptoms. Auscultation can be performed immediately, and I learned to listen not only to the chest but also to the neck.

It is essential to make integrated decisions based on both symptoms and physical findings.

For patients with chronic symptoms, even if they report that nothing has worsened, asking about what prompted the visit is important. I also learned the importance of addressing and following up on the patient’s main concern.

I felt that having a clear understanding of the rationale behind each interview question helps reduce the chances of missing important information. It is important to conduct interviews while keeping possible diagnoses in mind.

Showing the patients their test results during explanations helped them to understand and accept their current condition more easily. This showed me that visual, evidence-based explanations are important.

The physician first explained the current situation clearly and then presented the treatment plan, which seemed to facilitate the patient’s understanding. I would like to learn to do the same.

I should have asked more detailed questions regarding daily activities, such as the patient’s ability to walk. In addition, since amyloidosis had already been suspected, I regret not asking more specifically about symptoms, such as numbness in the extremities.

I learned the importance of gathering information independently and not relying solely on referral documents. Taking time to calmly organize information is essential.

I recognized the importance of explaining treatment risks and reviewing past medical and treatment histories thoroughly.

I would like to improve my ability to explain conditions clearly in patient-friendly language.
